# Behavioral and Biochemical Effects of *Mukia madrespatana* Following Single Immobilization Stress on Rats

**DOI:** 10.3390/medicina56070350

**Published:** 2020-07-14

**Authors:** Noreen Samad, Amna Ali, Farzana Yasmin, Riaz Ullah, Ahmed Bari

**Affiliations:** 1 Department of Biochemistry, Bahauddin Zakariya University, Multan 60800, Pakistan; amnaali394@gmail.com; 2 Department of Biomedical Engineering, NED University of Engineering and Technology, Karachi 75270, Pakistan; farzanayasminpk@yahoo.com; 3 Department of Food Engineering, NED University of Engineering and Technology, Karachi 75270, Pakistan; 4 Department of Pharmacognosy (MAPPRC), College of Pharmacy, King Saud University, Riyadh 12372, Saudi Arabia; rullah@ksu.edu.sa; 5 Central Laboratory, College of Pharmacy, King Saud University, Riyadh 12372, Saudi Arabia; abari@ksu.edu.sa

**Keywords:** *Mukia madrespatana*, oxidative stress, stress, memory, antioxidant enzymes

## Abstract

*Background and Objectives:* Elevated oxidative stress has been shown to play an important role in the diagnosis and prognosis of stress and memory-related complications. *Mukia madrespatana* (*M. madrespatana*) has been reported to have various biological and antioxidant properties. We intended to evaluate the effect of *M. madrespatana* peel on single immobilization stress-induced behavioral deficits and memory changes in rats. *Materials and Methods:* *M. madrespatana* peel (2000 mg/kg/day, orally) was administered to control and immobilize stressed animals for 4 weeks. Anxiolytic, antidepressant, and memory-enhancing effects of *M. madrespatana* were observed in both unstressed and stressed animals. *Results:* Lipid peroxidation was decreased while antioxidant enzymes were increased in both unstressed and stressed animals. Acetylcholine level was increased while acetylcholinesterase activity was decreased in both *M. madrespatana* treated unstressed and stressed rats. There was also an improvement in memory function. Serotonin neurotransmission was also regulated in *M. madrespatana* treated rats following immobilization stress with anxiolytic and anti-depressive effects. *Conclusion:* Based on the current study, it is suggested that *M. madrespatana* has strong antioxidant properties and may be beneficial as dietary supplementation in stress and memory-related conditions.

## 1. Introduction

Stress is a major threat to the body that alters homeostasis at the cellular level. The immobilization stress model is an easy and continent method for inducing both psychological and physical stresses in mammals [1]. Stress may lead to a variety of central nervous system irregularities that include anxiety, depression, learning, and memory functions [2]. According to earlier reports, there is restricted mobility, an anxiety-like effect, and deterioration of antioxidant enzyme activities following immobilization stress [3].

The activation of hypothalamic pituitary adrenal (HPA)-axis by a stress situation induced greater release of glucocorticoid, which damaged neurons and their functions [4]. The increased levels of glucocorticoid stimulated the release of glutamate in the cortex and limbic part of the brain [5]. The increased neuronal flow of glutamate can harm mitochondria and enhance cellular respiration [6]. This increase in cellular respiration generated additional free radicals, causing an unevenness between pro-oxidant and antioxidant defense system [7,8]. Free radicals resulted in oxidative harm at the cellular level and damaged cellular machinery [9]. The brain is a very sensitive organ because of its high content of readily oxidized fatty acids, high expenditure of oxygen, and low antioxidant levels [10]. Additionally, free radicals are produced constantly as an entailment of normal metabolic functions. The increased free radicals’ production and weak antioxidant defense system have been associated with serious neurological ailments including depression, dementia, Alzheimer’s disease, etc. However, enhancement in the antioxidant defense system can combat the increased free radical production and control/protect the body from a variety of threats [11,12].

Extensive studies reported that natural antioxidants may be useful against stress-instigated neuronal complications [13,14]. Medicinal plants play a vital role in therapeutics. There is a growing interest in plant-based therapy from a few years back due to its natural origin and minimum adverse effects [15,16]. At present, clinical trials are underway to evaluate the protective effects of medicinal plants against neurological disorders, i.e., depression, Alzheimer’s, Parkinsonism, etc. Biologically-active components present in the medicinal plants made them useful for treating various diseases. Bio-activity of the plants having medicinal potential is related to bioactive components, i.e., alkaloids, flavonoids, polyphenols, tannins, triterpenoids, coumarins, and glycosides [14,15,16].

*Mukia madrespatana* (*M. madrespatana*) (L.) M. Roem. (Cucurbitaceae), which is commonly known as the Madras pea pumpkin, is one such plant [17], with distribution throughout the tropics and subtropics of the Old World [18]. Phytochemistry of *M. madrespatana* discovered the presence of phenolic, tannins, flavonoids, alkaloids, and saponins. These phytochemical compounds in *M. madrespatana* are strong-reducing agents due to their abundant OH groups that enhance their antioxidant activity [19]. Various in-vitro and in-vivo studies have been conducted with the *M. madrespatana* plant. It was reported that it has anti-inflammatory, antiarthritic [20], anti-asthmatic, antitussive, antihistaminic, anti-bronchitic [21], antihypertensive, and anti-diabetic [22] effects due to its powerful antioxidant potential. 

Taking into consideration in the first part of the present study, various doses of *M. madrespatana* peel powder were used to evaluate the exploratory activity of rats in the novel and familiar environment, via various behavioral analyses, to select a potential dose for further experiments. In the second part of the study, anti-stress, antioxidant, and memory-enhancing effects of the selected dose of *M. madrespatana* peel powder were evaluated following acute immobilization stress. Before starting the experimental procedure, acute toxicity of *M. madrespatana* in animals was monitored.

## 2. Materials and Methods

### 2.1. Animals

In the present work, adult male Albino Wistar rats (weighing 160–180 gm, eight weeks old) were locally bred and utilized in this study. Rats were housed in an individual cage (with static racks) to keep away from the social contact effect because social contact can influence behavioral analysis of animals. The Albino Wistar rats were given a standard rodent diet (A control diet (4.47 kcal/g) containing 25% fat, 50% carbohydrate, and 25% protein [23]) and drinking water under a controlled temperature (20 ± 5 °C) and a 12-hour light/dark cycle. Before starting the experimental work, rats experienced seven days of acclimation time and different conduct measures to diminish the stress of newness and treatment. The Institutional Ethical Committee (Ref # D-1991/1-Biochem; Dated: September 29, 2018) approved all experiments. Additionally, all experiments were performed in strict accordance with the National Institute of Health Guide for Care and Use of Laboratory Animals (Publication No. 85-23, revised 1985).

### 2.2. Plant Material and Chemicals 

Fresh fruits of *Mukia maderaspatana* (*M. madrespatana*) were collected from the area of the field near Multan City, Pakistan. The plant material was recognized and authenticated by the taxonomist (Dr. Zafar Ullah Zafar, Department of Botany, Bahauddin Zakariya University, Multan) and a voucher (R.R. Stewart F.W. Pak.706 (3)) was retained in an herbarium. Fresh fruits of *M. madrespatana* were washed and peeled off. Peel of *M. madrespatana* was air-dried at room temperature and then ground into a fine powder using an electric grinder and stored in an airtight container. The selection of low and high doses of *M. madrespatana* fruit peel powder in the present study based on the previous report in which the whole plant of *M. madrespatana* with various extracts (aqueous and ethanolic) was used with various dosages, i.e., 100–200 mg/kg body weight [24] and 2000 mg/kg body weight [25,26]. The required amount of powder was weighed and mixed well in deionized water (3 mL) and fed to animals by the gavage technique. Every time a fresh drug dose was prepared for oral administration. All chemicals used in the current study were purchased from Chemical Co. Sigma-Aldrich, St. Louis, MO, USA.

### 2.3. Acute Toxicity Test

The toxicity procedure was performed as reported previously [27]. Twelve animals (*n* = 4) were used for the acute toxicity test as reported previously [28]. Two sets of rats (*n* = 4) were accommodated overnight in a fasting condition with tap water and food was withdrawn for 4–5 h after oral intake of *M. madrespatana* fruit peel powder. Starting a dose of 125 mg/kg body weight was given to the first set and a maximum dose of 2000 mg/kg body weight was given to the second set. The third set of rats (*n* = 4) was drinking tap water and all the rodents were observed individually. Clinical signs were examined. Body posture, tremors, locomotion, pain response, vocalization, body weight, water intake, etc. were also observed. No death was seen with given doses. According to Economic Co-operation and Development (OECD) guidelines, the LD50 value considered above 125 mg/kg and 2000 mg/kg body weight.

### 2.4. Experimental Protocol

#### 2.4.1. Investigation 1: Effect of Various Doses of *M. madrespatana* to Assess the Exploratory Activity in the Novel and Familiar Environment to Find Out the Potential Dose

Thirty animals were housed individually and divided into six groups each (*n* = 5). Various doses of *M. madrespatana* fruit peel powder ranging from 0.0, 125.0, 250.0, 500.0, 1000.0, and 2000.0 mg/kg/day were orally given to each group at 9:00–10:00 a.m. for 28 days. During this time, the health of the animals was monitored. After four weeks of the home cage (familiar environment) and open field (novel environment), activity was performed for 5 min each.

#### 2.4.2. Investigation 2: Effect of Potential/Selected Dose (2000 mg/kg/day) of *M. madrespatana* on Behavioral Deficits, Oxidative Stress, and Memory Function Induced by Single Immobilization Stress

20 rats separated into two groups (*n* = 10). (i) Control group and (ii) test group. The control group received drinking water while the test group was given 2000 mg/kg/day of M.M. fruit peel powder orally (at 10:00–11:00 a.m.) for four weeks. On day 29, both groups were further divided into four groups (*n* = 5) (i) Unstressed + water (ii) Stressed + Water (iii) Unstressed + M.M (iv) Stressed + M.M. Number of animals in sub-groups used in the present study was already reported [28]. Animals of the stressed groups were immobilized on metal wire grids for 2 h (at 7:00–9:00 am) in a separate room. Meanwhile, unstressed groups were left in their home cages. For 24 h post-immobilization stress (on day 30), behavioral activities include: (i) an elevated plus maze (EPM) test, (ii) light/dark activity (LDA) test, (iii) forced swim test (FST), and (iv) Morris water maze (MWM) test (acquisition and short term memory) were conducted between 9:00 a.m. to 6:00 p.m. with the help of blind observers. On day 31, long-term memory of all animals was assessed using MWM. After that, animals were decapitated and the hippocampi (brain region) were removed [29], kept frozen at −20 °C, and utilized for further biochemical analysis 24 h after the decapitation.

#### 2.4.3. Immobilization Stress Procedure

The animals were immobilized on wire grids of 10″ × 9″ fitted with a Perspex plate of 9″ × 6.5″. The previously described procedure was used for the immobilization of animals [30]. Immobilization was produced by pressing the forelegs of the rat through the gaps in the metal grids and taping them together with Zinc Oxide plaster tape. Hind limbs were also taped and the head of the animal rested on the Perspex plate. Unstressed groups were kept in their home cages during the immobilization period.

### 2.5. Behavioral Tests

#### 2.5.1. Elevated Plus Maze (EPM) Test

Elevated plus-maze is used for the measurement of anxiolytic activity of test compounds and, for the said purpose we used, the apparatus of plus shape with the total dimensions of 110 × 10 × 15 cm. The closed and open arms are distinguished with the central 10 cm arena. For the testing purpose, we used the protocol established previously [31]. The animal was permitted to visit on the elevated stage for 5 min and behavioral assessment was done by its time and frequency spent in open arm vs. closed arm. The top-mounted camera system (Logitech C-310, Basel, Switzerland) recorded the videos. Between each trial, the apparatus was cleaned with 70% isopropyl alcohol to wash out the smell of the previous trial.

#### 2.5.2. Light-Dark Activity (LDA) Test

A customized-made apparatus of acrylic glass was used for measuring an animal’s anxiety behavior in a two compartmental system of light and dark environment. The dimension of the apparatus is 40 × 30 × 35 cm where light and dark compartments are equally divided, i.e., 20 cm each and central door of 10 × 10 cm allows free passage of animals between the compartments. The total duration of the test is 5 min in which the duration of the animal’s time spent in each compartment was noted with the lateral camera recording system. Subsequently, the recorded videos were analyzed by a blinded observer to the experiments with the help of a stopwatch.

#### 2.5.3. Forced Swim Test (FST)

The forced swim test (FST) has a widely employed method for evaluating the depressant/antidepressant behavior of rodents [31]. In the experiment, we used a glass tank with a dimension of 45 cm in height and 30 cm in the radius for assessing the antidepressant activity of the test substances. The apparatus was full of water (25 °C) up to ~35 cm where the feet of the animal should not get the support from the basement and become, simultaneously, inescapable from the tank [28]. In a trial session of 15 min each, all animals were allowed to force swim. In an experimental session, 24 h after the trial session, the treated rats were challenged to the environment of inescapability and difference between the states of mobility (climbing and swimming) vs. immobility was observed by lateral recording, which was, subsequently, analyzed by a blinded observer. The rat will be considered in a phase of depression where it spent most of the time in a phase of immobility and makes virtually no efforts to escape and merely attempt to retain its head above the water.

#### 2.5.4. Morris Water Maze (MWM) Test

In this test, we assessed the impact of M.M. fruit peel powder on the retention of spatial memory in rats. For the purpose, we used a customized circular tank of grey color with a dimension of 150 cm in radius and 50 cm in height [32]. The tank was filled with water and the temperature was maintained at 25 ℃ throughout the experiment. The tank was equally divided into quadrants, i.e., north-east (NE), south-east (SE), (north-west (NW), and south-west (SW), and an opaque color platform made of acrylic glass was placed in the SW quadrant of the tank. The distal cues with distinct shapes were placed in all four quadrants of tank walls, which were helpful for the animal to navigate in the tank and direction. Before starting the experiment, the color of the water was changed to murky with the addition of nonhazardous white ink and the platform was submerged 1–2 cm in water. The test was performed in two sessions: (1) the training session and (2) test phases, which is comprised of retention of short (1 h after training) and long (24 h after training) term retention of memory [33]. The animal was placed all the time from the same quadrant, i.e., NE and headed for the wall of the tank. In the training phase, each rat was allowed 2 min time to navigate and find the hidden platform and, if located within the cut-off time, the animal was permissible to stay at the platform for 10–20 s to navigate the surroundings. Otherwise, after 2 min, the animal was directed to the platform by manually guiding it to the platform. One hour after the training phase, short-term retention of memory was performed on the trained rats, and latency to reach the platform was noted. The videos were recorded on the top-mounted camera and, subsequently, analyzed by Any-Maze (V 6.01) video tracking software (Wood Dale, IL, USA).

### 2.6. Biochemical Analysis

Decapitation of all animals was done on the same day after behavioral tests. Brain samples were detached and the hippocampus was removed, weighed (averagely 76 mg), and washed with 0.9% saline solution and used for various biochemical tests 24 h after the decapitation. A tissue homogenate 10% (wt./vol.) was prepared with phosphate buffer (0.1 M, pH 7.4) and got by centrifugation (12,000× *g*) for 20 min at 4 °C for all biochemical estimations.

#### 2.6.1. Determination of Malondialdehyde (MDA)

The method of Chow & Tappel [34] was used for the analysis of malondialdehyde with some changes in the procedure. 0.3 µL hippocampus homogenate was added in a 2 mL trichloro-acetic acid-thio-barbituric acid (TCA-TBA) solution and then boiled in a water bath for 20 min. The mixture was then cooled down and centrifuged (35,000 RPM) for 10 min. Light pink-colored supernatant was collected and 532 nm absorbance was used to note the absorbance.

#### 2.6.2. Determination of Superoxide Dismutase (SOD) Activity

The previously reported method was used for the analysis of SOD [35]. A total of 300 µL of hippocampus homogenate was centrifuged with 0.75 mL ethanol and 0.15 mL of chloroform. After centrifugation, 500 µL supernatant was taken and then 0.5 mL EDTA and 1.0 mL of 0.1 M carbonate-bicarbonate buffer (pH 10.2) were added. By adding 0.5 mL of an epinephrine reaction, absorbance was taken at 480 nm, and the percentage inhibition of SOD was calculated.

#### 2.6.3. Determination of Catalase (CAT) Activity

A total of 100 µL hippocampus supernatants mixed with 1.4 mL solution comprised of 400 µL of hydrogen peroxide and 1 mL of phosphate buffer (0.01 M, pH 7.0). The reaction was stopped 1 min after by adding 2 mL of the reagent (dichromate acetic acid). To calculate the activity of CAT, absorbance was taken at 620 nm, as described previously [36].

#### 2.6.4. Determination of Glutathione Peroxidase (GPx) Activity

The method of Flohe & Gunzler [37] was used to measure the activity of GPx as µmol/min/g of the hippocampus. A total of 1.0 mL of reaction solution that contained 0.3 mL phosphate buffer (0.1 M, pH 7.4), 0.2 mL of glutathione reduced, 0.1 mL of sodium azide, 0.1 mL of hydrogen peroxide, and 0.3 mL of homogenate was prepared. The reaction solution was incubated for 15 min at 37 °C by adding 0.5 mL of TCA. The reaction was terminated and the solution was centrifuged (1500× *g*) for 15 min and filtrate was removed. A total of 0.2 mL phosphate buffer and 0.7 mL 5,5’-dithiobis-(2-nitrobenzoic acid (DTNB) were mixed with 0.1 mL of filtrate. The absorbance of the solution was recorded at 420 nm.

#### 2.6.5. Determination of Acetylcholinesterase (AChE) Activity

The method of Ellman et al. [38] was used to estimate the activity of AChE. A total of 0.4 mL hippocampus homogenate, 2.6 mL phosphate buffer, and 0.1 mL DTNB were mixed by bubbling air and put down into a spectrophotometer. When the reaction solution was steady, the basal reading followed by the addition of 5.2 µL of acetylthiocholine iodide in a cuvette contained a reaction solution. Absorbance was recorded at 412 nm. The activity of AChE was mentioned as µmol/min/g of tissue.

#### 2.6.6. Determination of 5-HT and 5-HIAA

5-hydroxy indole acetic acid (5-HIAA) and 5-hydroxytryptamine (5-HT) levels in the hippocampus were estimated using the method described by Samad et al. [28]. Reversed-phase High-Performance Liquid Chromatography (HPLC) with an electrochemical detector (Shimadzu LEC 6A detector) was performed to detect levels of biogenic amines in brain samples. The electro-chemical (EC) detector was operated at a potential of +0.8 V. The stationary phase used for separation is a 5-μ Shim-pack octa-decyl silane (ODS) column having an internal diameter of 4.0 mm and a length of 150 mm. The mobile phase that passes through a column with a pump pressure of 2000–3000 psi contains octyl sodium sulfate (0.023%) in 0.1 M phosphate buffer at pH 2.9.

#### 2.6.7. Determination of Acetylcholine (ACh)

The level of ACh concentration was determined as described by Reference [39] and was presented as µmol/g of the hippocampus. The tissue sample was boiled to release the bound ACh and inactivate the enzyme. The reaction mixture was then mixed with ferric chloride 1% (1000 μL) to form a brown color complex and read at 540 nm against the reagent blank.

### 2.7. Statistical Analysis

Data of exploratory activity in the novel and familiar environment were analyzed by One-Way ANOVA. All the biochemical and behavioral (Post stress) data were analyzed by Tukey’s test and this was followed by two-way ANOVA. All the data analyzed by SPSS ver. 20.0 (IBM, Chicago, IL, USA). *p* < 0.05 was taken as significant.

## 3. Results

### 3.1. Various Doses of M. madrespatana Peel

**Figure 1:**
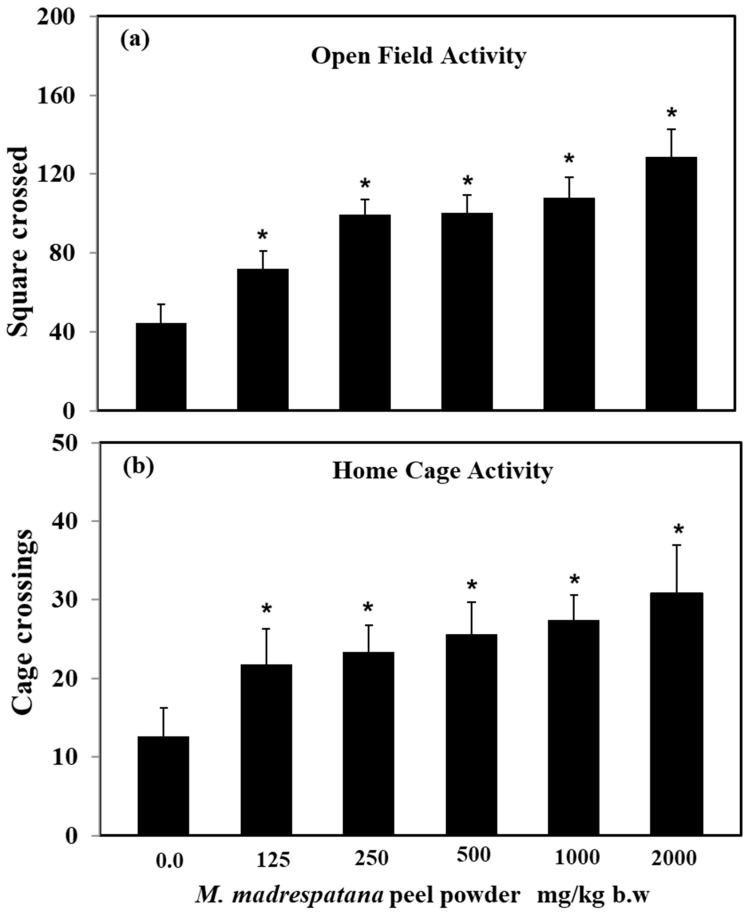
Effects of M. *madrespatana* with various doses in a novel (**a**) and familiar (**b**) environment. Values are means ± standard deviation (*n* = 5) 4 weeks after the administration of the drug. Significant differences by Tukey’s test * *p* < 0.05 from control animals following one-way ANOVA.

Effect of *M. madrespatana* doses on exploratory activities in an open field and home cage shown in Figure 1. Data on activity in an open field test was evaluated by one-way ANOVA (df = 5.24) (F = 87.45, *p* = 0.001), which showed a substantial effect of *M. madrespatana.* Activity of *M. madrespatana* at doses 0.0, 125.0, 250.0, 500.0, 1000.0, and 2000.0 mg/kg/day substantially increased when evaluated by Tukey’s test.

Data on activity in the home cage was analyzed by one-way ANOVA (df = 5.24) (F = 50.72, *p* = 0.001) showed substantial effect. *M. madrespatana* at doses 0.0, 125.0, 250.0, 500.0, 1000.0, and 2000.0 mg/kg/day substantially increased when evaluated by Tukey’s test.

### 3.2. Elevated Plus-Maze (EPM)

**Figure 2:**
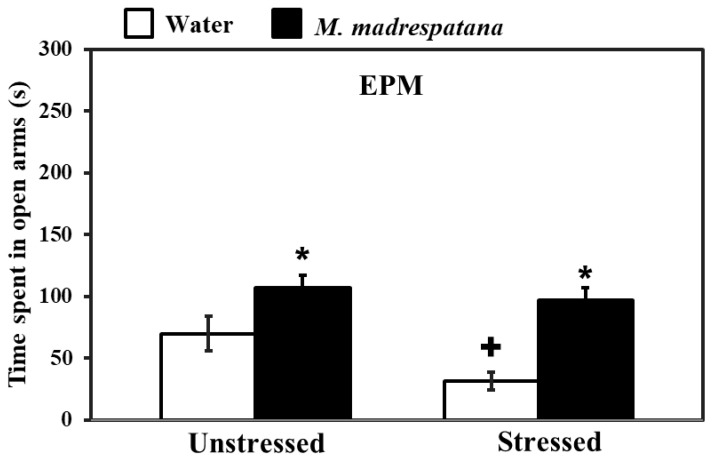
Effects of administration of *M. madrespatana* on the anxiety profile in unstressed and stressed rats observed in EPM. Values are mean ± standard deviation (*n* = 5). Data were analyzed by Tukey’s test following two-way ANOVA. Statistical difference is expressed as * *p* < 0.05 versus respective control and + *p* < 0.05 versus unstressed rats.

Figure 2 shows the effect of *M. madrespatana* on anxiety-like symptoms tested via EPM. Data evaluated by two-way ANOVA (df = 1.16) showed the substantial effects of stress (F = 26.192, *p* = 0.001), *M. madrespatana* (F = 118.678, *p* = 0.0001), and interaction between stress × *M. madrespatana*. (F = 8.966, *p* = 0.001). Tukey’s test exhibited that single stress lessened time spent in an open arm. Time spent in open arms markedly enhanced in *M. madrespatana* treated (unstressed and stressed) than their respective control.

### 3.3. Light/Dark Box Activity

**Figure 3:**
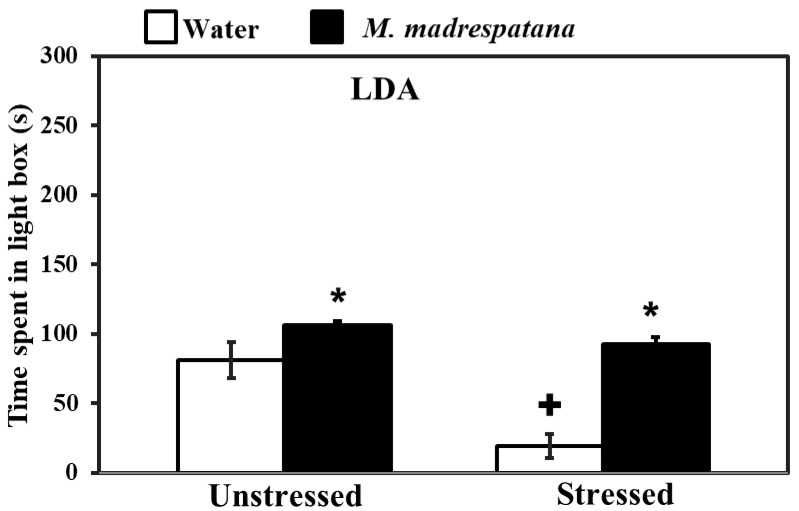
Effects of administration of M.M. on anxiety profile in unstressed and stressed rats observed in the light-dark activity box. Values are mean ± standard deviation (*n* = 5). Data were analyzed by Tukey’s test following two-way ANOVA. Statistical difference is expressed as * *p* < 0.05 versus respective control and + *p* < 0.05 versus unstressed rats.

Figure 3 shows the effect of *M. madrespatana* on anxiety-like behavior tested via the light/dark box. Data evaluated by two-way ANOVA (df = 1.16) showed the substantial effects of stress (F = 105.527, *p* = 0.0001), *M. madrespatana* (F = 181.696, *p* = 0.0001), and interaction between stress × *M. madrespatana* (F = 42.541, *p* = 0.002). Tukey’s test exhibited that acute stress lessened time spent in the lightbox. Time spent in the lightbox substantially enhanced treated unstressed and stressed animals in *M. madrespatana* than their counterparts.

### 3.4. Forced Swim Test (FST)

**Figure 4:**
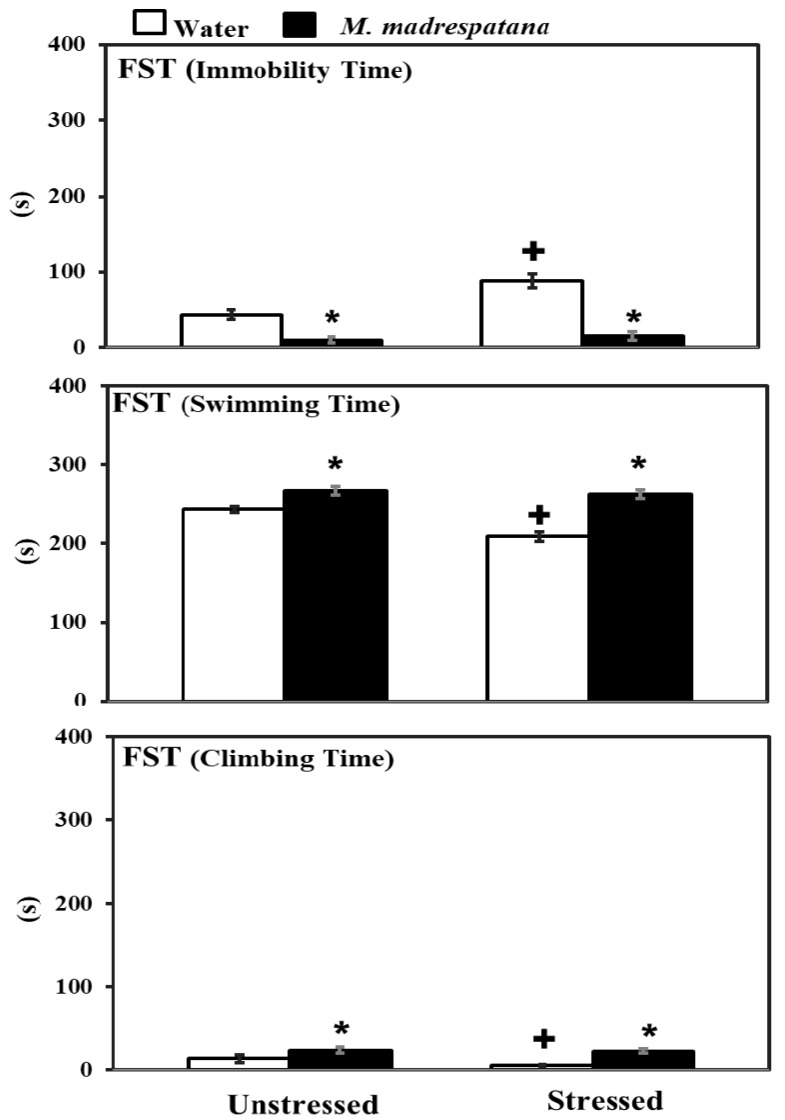
Effects of *M. madrespatana* administration following immobilization stress on immobility, swimming, and climbing time in FST. Values are mean ± standard deviation (*n* = 5). Data were analyzed by Tukey’s test following two-way ANOVA. Statistical difference is expressed as * *p* < 0.05 versus respective control and + *p* < 0.05 versus unstressed rats.

Figure 4 shows the effects of *M. madrespatana* on depression-like symptoms tested through FST. Data evaluated by two-way ANOVA (df = 1.16) showed the substantial effects of stress (F = 72.129, *p* = 0.001), *M. madrespatana* (F = 329.089, *p* = 0.0001), and stress × *M. madrespatana* (F = 45.244, *p* = 0.001) on immobility time. Tukey’s test exhibited that immobilization substantially enhanced immobility time in water-treated rats. Immobility time substantially reduced in *M. madrespatana*-treated unstressed and stressed animals than their counterparts.

Data evaluated by two-way ANOVA (df = 1.16) showed the substantial effects of stress (F = 59.03, *p* = 0.001), *M. madrespatana* (F = 233.71, *P* = 0.0001), and stress × *M. madrespatana* (F = 34.35, *p* = 0.001) on swimming time. Tukey’s test exhibited that immobilization substantially reduced swimming time in water-treated rats. Swimming time substantially enhanced in *M. madrespatana* treated unstressed and stressed animals than their counterparts.

Data evaluated by two-way ANOVA (df = 1.16) showed the substantial effects of stress (F = 8.78, *p* = 0.009), *M. madrespatana* (F = 83.88, *p* = 0.0001), and stress × *M. madrespatana* (F = 4.64, *p* = 0.04) on climbing time. Tukey’s test exhibited that immobilization substantially reduced climbing time in water-treated rats. Climbing time substantially enhanced in *M. madrespatana* treated unstressed and stressed animals than their counterparts.

### 3.5. Morris Water Maze (MWM) Test

**Figure 5:**
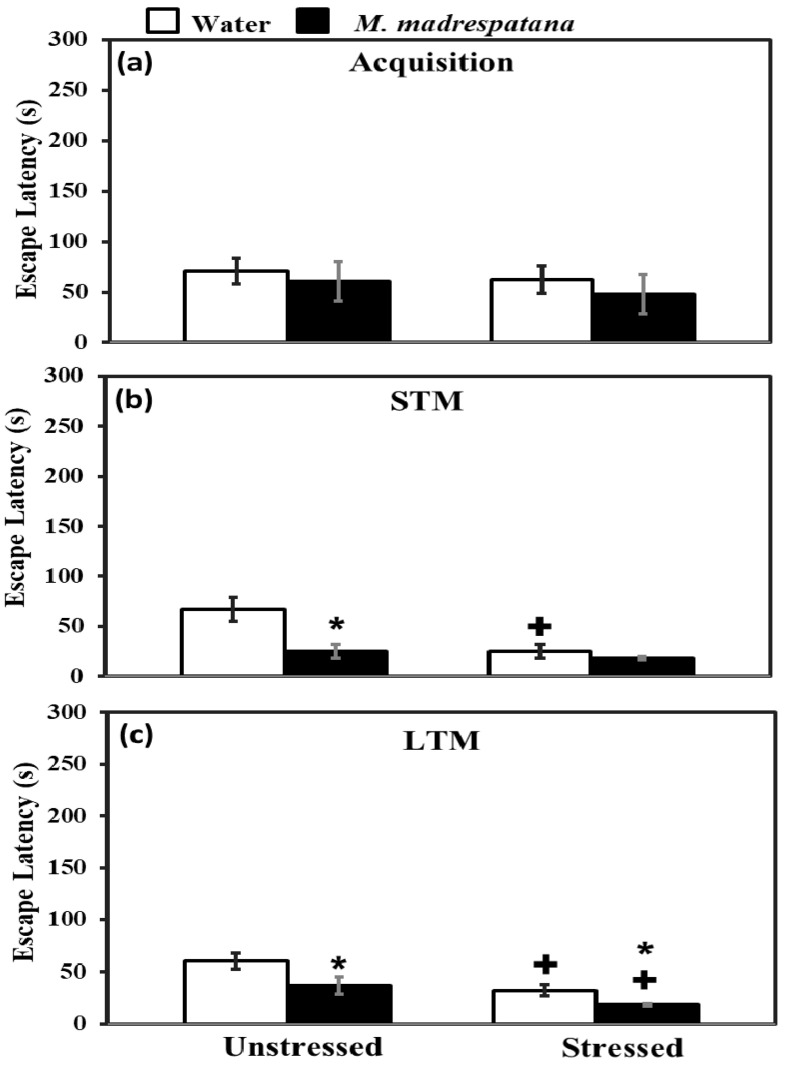
Effect of *M. madrespatana* administration following single stress on acquisition (**a**) STM (**b**) and LTM (**c**) in terms of escape latency (s) assessed by MWM. Values are mean ± standard deviation (*n* = 5). Data were analyzed by Tukey’s test following two-way ANOVA. Statistical difference is expressed as * *p* < 0.05 versus respective control and + *p* < 0.05 versus unstressed animals.

Figure 5 shows the effect of *M. madrespatana* on memory tests via the Morris water maze. All the data evaluated by two-way ANOVA (df = 1.16). Data of acquisition (5a) exhibited that effect of stress (F = 2.092, *p* = 0.11), *M. madrespatana* (F = 3.329, *p* = 0.14), and stress × *M. madrespatana* (F = 0.087, *p* = 0.21) was non-substantial.

Data on short term memory (STM) (5b) exhibited a substantial effect of stress (F = 14.381, *p* = 0.005), *M. madrespatana* (F = 128.833, *p* = 0.0001), and stress × *M. madrespatana* (F = 58.285, *p* = 0.001). Tukey’s test exhibited a considerable decrease in escape latency in M.M. treated unstressed and control stressed groups than their respective control group.

Data on long-term memory (LTM) (5c) exhibited a substantial effect of stress (F = 69.992, *p* = 0.0001) and *M. madrespatana* (F = 44.026, *p* = 0.001) while stress × *M. madrespatana* interaction (F = 2.818, *p* = 0.02) was non-substantial. Tukey’s test showed a considerable decrease in escape latency of *M. madrespatana*-treated unstressed and stressed groups than their counterparts. Single immobilization stress also lessened escape latency in water-treated animals. Escape latency was smaller in *M. madrespatana*-treated stressed animals than unstressed animals.

### 3.6. Assessment of Oxidative Stress

**Figure 6:**
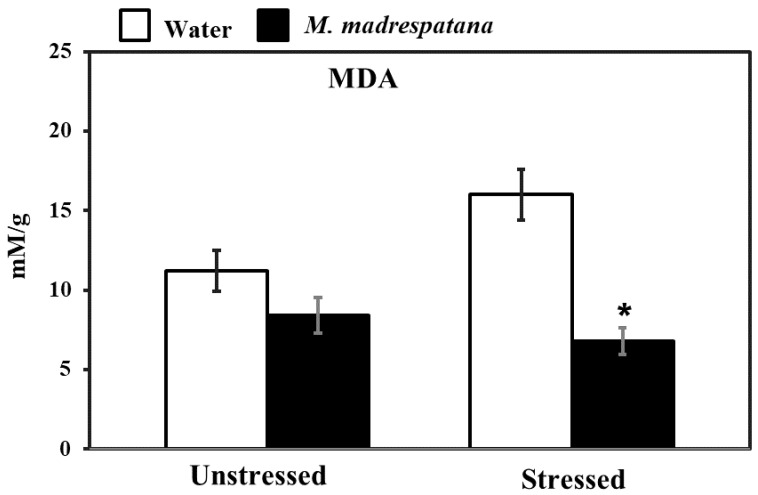
Effects of *M. madrespatana* administration following immobilization stress on hippocampal MDA activity. Values are mean ± standard deviation (*n* = 5). Data were analyzed by Tukey’s test following two-way ANOVA. Statistical difference is expressed as * *p* < 0.05 versus respective control and + *p* < 0.05 versus unstressed animals.

Figure 6 shows the effect of *M. madrespatana* on malondialdehyde (MDA) content of the hippocampus in unstressed and stressed animals. Data evaluated by two-way ANOVA (df = 1.16) showed substantial effects of *M. madrespatana* (F = 116.12, *p* = 0.001), stress (F = 8.25, *p* = 0.03), and stress × *M. madrespatana* (F = 33.03, *p* = 0.004). Tukey’s test exhibited that the contents of MDA markedly increased following immobilization in water-treated animals. The contents of MDA reduced in *M. madrespatana*-treated unstressed and stressed rats than their respective control.

### 3.7. Determination of Antioxidant Enzymes

**Figure 7:**
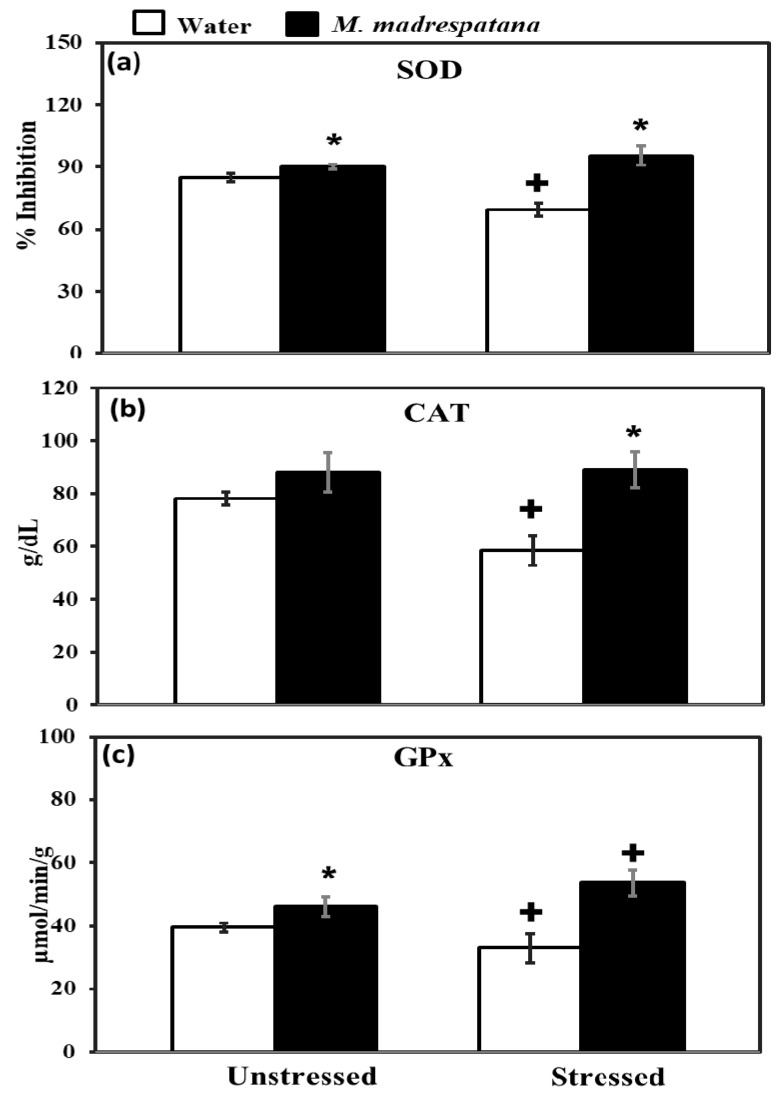
Effect of *M. madrespatana* administration following immobilization stress on hippocampal SOD (**a**), CAT (**b**), and GPx (**c**) activity. Values are mean ± standard deviation (*n* = 5). Data was analyzed by Tukey’s test following two-way ANOVA. Statistical difference is expressed as * *p* < 0.05 versus respective control and + *p* < 0.05 versus unstressed rats.

Figure 7 shows the effect of *M. madrespatana* on antioxidant enzyme levels in the hippocampus of unstressed and stressed animals. All the data evaluated by two-way ANOVA (df = 1.16). Data on SOD exhibited a substantial effect of stress (F = 14.381, *p* = 0.01), *M. madrespatana* (F = 128.833, *p* = 0.001), and stress × *M. madrespatana* (F = 58.285, *p* = 0.0004). Tukey’s test exhibited that the activity of SOD in the hippocampus increased in M.M treated unstressed and stressed rats than their counterparts. SOD levels lessened in water + stressed than water + unstressed group.

Data on CAT showed a substantial effect of stress (F = 12.18, *p* = 0.005), *M. madrespatana* (F = 56.95, *p* = 0.002), and stress × *M. madrespatana* (F = 15.01, *p* = 0.01). Tukey’s test exhibited that CAT activity in the hippocampus was decreased in control stress. The activity of CAT was enhanced in *M. madrespatana*-treated stressed animals than water-treated stressed animals.

Data on GPx activity showed substantial effects of *M. madrespatana* (F = 75.985, *p* = 0.0001) and interaction of stress × *M. madrespatana* (F = 20.104, *p* = 0.001) while the effect of stress (F = 0.106, *p* = 0.13) was non-substantial. Tukey’s test showed GPx activity in the hippocampus increased *M. madrespatana*-treated unstressed and stressed versus their counterparts. On the other hand, single stress lessened GPx activity in water-treated animals.

### 3.8. Determination of AChE

**Figure 8:**
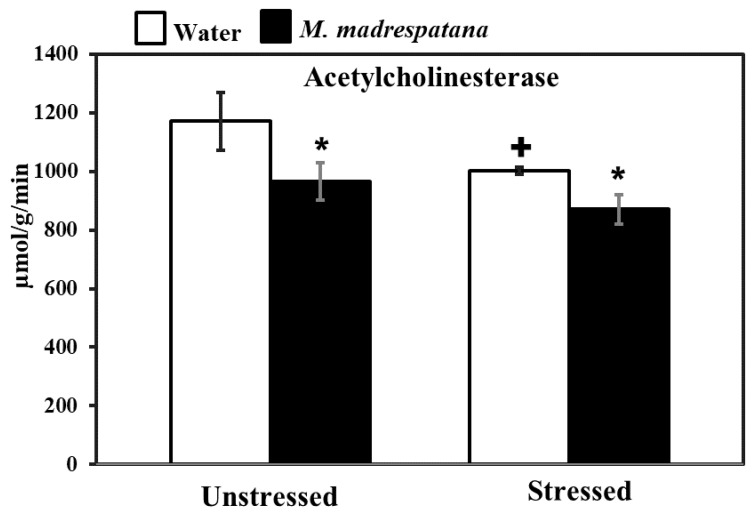
Effects of M.M. administration following immobilization stress on AChE activity in the hippocampus. Values are mean ± standard deviation (*n* = 5). Data were analyzed by Tukey’s test following two-way ANOVA. Statistical difference is expressed as * *p* < 0.05 versus respective control and + *p* < 0.05 versus unstressed animals.

Figure 8 shows the effect of *M. madrespatana* on hippocampal AChE activity in unstressed and stressed animals. Data evaluated by two-way ANOVA exhibited a substantial effect of stress (F = 21.482, *p* = 0.0003), *M. madrespatana* (F = 34.962, *p* = 0.0001), and stress × *M. madrespatana* (F = 1.706, *p* = 0.01). Tukey’s test exhibited single stress lessened AChE activity in control animals. The activity of AChE significantly lessened in *M. madrespatana*-treated unstressed rats and stressed rats than their respective control.

### 3.9. Determination of 5-Hydroxy Tryptamine (5-HT) and 5-Hydroxy Indole Acetic Acid (5-HIAA) Levels

**Figure 9:**
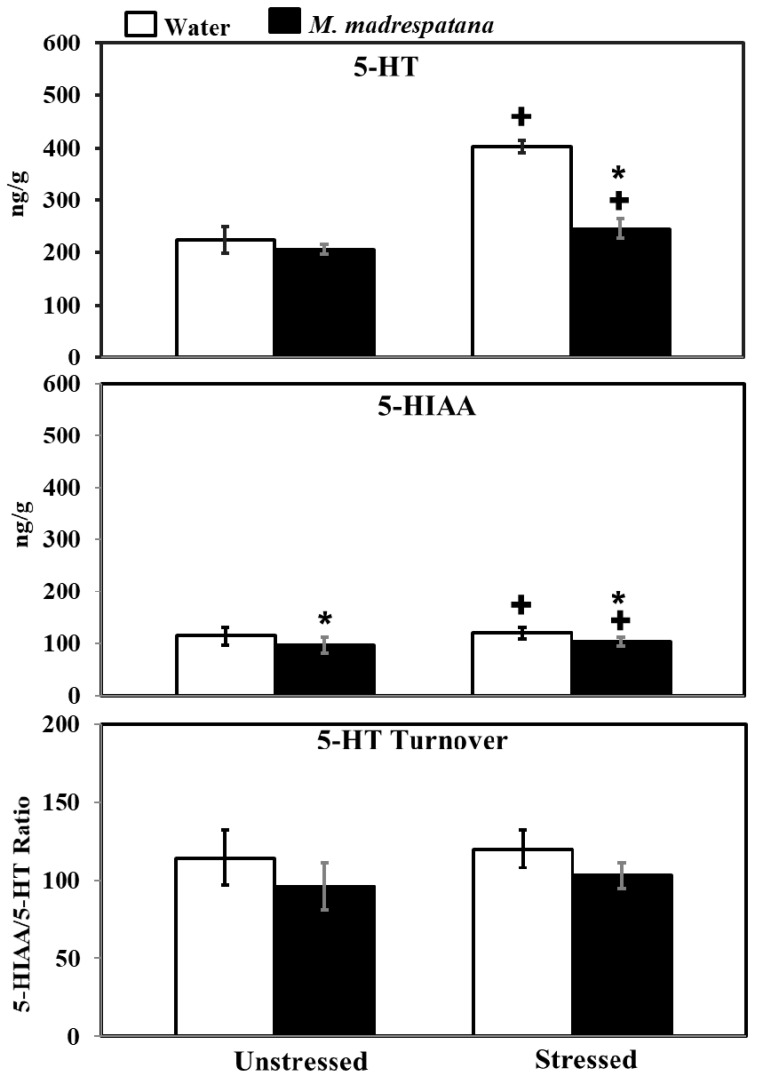
Effect of *M. madrespatana* administration following immobilization stress on 5-HT, 5-HIAA levels, and 5-HT turnover in terms of 5-HIAA/5-HT ratio in the hippocampus. Values are mean ± standard deviation (*n* = 5). Data were analyzed by Tukey’s test following two-way ANOVA. Statistical difference is expressed as * *p* < 0.05 versus respective control and + *p* < 0.05 versus unstressed animals.

Figure 9 shows the effect of *M. madrespatana* on 5-HT and 5-HIAA levels of the hippocampus in unstressed and stressed rats. All the data evaluated by two-way ANOVA (df = 1.16). Data on 5-HT levels exhibited a substantial effect of stress (F = 188.855, *p* = 0.0001), *M. madrespatana* (F = 123.386, *p* = 0.0001) stress × *M. madrespatana* (F = 77.935, *p* = 0.002). Tukey’s test showed that 5-HT levels of the hippocampus significantly enhanced in control stressed subjects than unstressed subjects. The levels of 5-HT in the hippocampus decreased in *M. madrespatana*-treated stressed animals than their respective control. While in *M. madrespatana*-treated stressed animals, levels of 5-HT were greater than *M. madrespatana*-treated unstressed rats.

Data on 5-HIAA levels exhibited a substantial effect of stress (F = 455.027, *p* = 0.0001), *M. madrespatana* (F = 556.172, *p* = 0.0001), and interaction between stress × *M. madrespatana* (F = 200.325, *p* = 0.0003). Tukey’s test showed that single stress increased 5-HIAA levels of the hippocampus in water-treated animals. The levels of 5-HIAA decreased in both *M. madrespatana*-treated unstressed and stressed animals. The levels of 5-HIAA greater in *M. madrespatana*-treated stressed than unstressed animals.

Data on 5-HIAA/5-HT ratio exhibited a substantial effect of *M. madrespatana* (F = 12.32, *p* = 0.003), while non-substantial stress (F = 1.525, *p* = 0.235) and interaction between stress × *M. madrespatana* (F = 0.023, *p* = 0.881). Tukey’s test showed non-substantial effects.

### 3.10. Determination of ACh Levels

**Figure 10:**
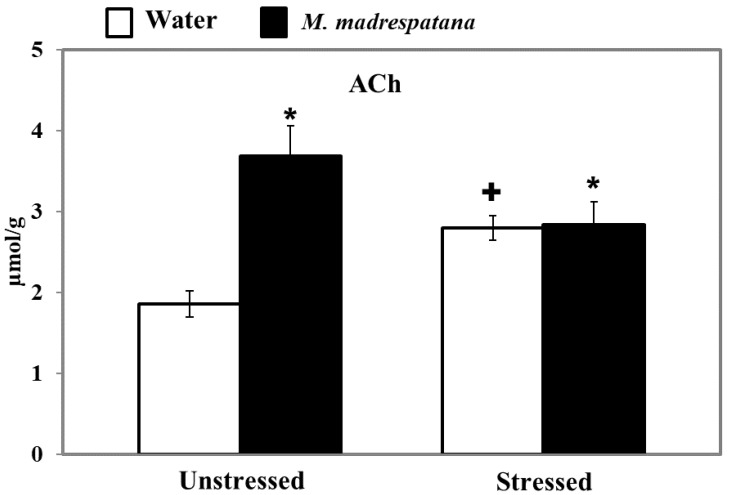
Effect of *M. madrespatana* administration following immobilization stress on ACh levels in the hippocampus. Values are mean ± SD (*n* = 5). Data were analyzed by Tukey’s test following two-way ANOVA. Statistical difference is expressed as * *p* < 0.05 versus respective control and + *p* < 0.05 versus unstressed animals.

Figure 10 displays the effect of *M. madrespatana* on ACh levels of the hippocampus in unstressed and stressed rats. All the data evaluated by two-way ANOVA (df = 1.16). Data on ACh levels exhibited a substantial effect of *M. madrespatana* (F = 61.12, *p* = 0.0001), stress × *M. madrespatana* (F = 55.97, *p* = 0.0001), while the effect of stress (F = 0.17, *p* = 0.680) was not substantial. Tukey’s test showed that ACh levels of the hippocampus significantly increased in the stressed control than the unstressed sample. The levels of ACh in the hippocampus increased in *M. madrespatana*-treated unstressed and stressed rats than their respective control.

## 4. Discussion

The present study aimed to investigate the effect of repeated administration of *M. madrespatana* fruit peel powder on single immobilization-induced behavioral and biochemical changes in the rat brain (brain region). The first of the study showed that normal (unstressed) animals increased exploratory activity dose-dependently. In the present study, psychological deficits and cognitive alteration disrupted serotonin neurotransmission and imbalanced oxidant-antioxidant levels were found in immobilized rats. Acute immobilization stress significantly decreased the time spent in open arm and lightbox in EPM and LDA, respectively, which indicates anxiety-like symptoms. The increased immobility time decreased swimming, and climbing time in FST following immobilization is representing depression-like behavior. Escape latency reduced after 60 min and 24 h of training in the MWM task indicating acute stress situations improve cognitive behavior. The anxiolytic, antidepressant, and improved memory functions observed in animals treated with a selected dose of *M. madrespatana* fruit peel powder (2000 mg/kg/day). The findings are related to the attenuation of immobilized stress-induced increased lipid peroxidation and decreased antioxidant mechanism by *M. madrespatana.* Levels of ACh in the hippocampus increased while the activity of AChE significantly decreased by *M. madrespatana*, which indicates cognition improvement. After single immobilization stress, normalization in serotonin metabolism by *M. madrespatana* suggests anti-stress property of *M. madrespatana*.

Stress-related psychiatric illnesses are evident as the malfunction of the antioxidant system. Oxidative stress plays a major role in the development of stress-induced behavioral deficits (anxiety and depression) in both human models [40] and experimental animal models [41,42]. An uncontrollable stress situation produces behavioral, neurochemical, and biochemical impairment [30,31]. The present study shows that immobilization stress-induced behavioral deficits such as depression-like symptoms measured by FST, anxiety-like behavior measured by LDA/EPM, and memory impairment (STM and LTM) measured by MWM tests. Behavioral decline (anxiety and depression) and cognitive impairment are the significant features of stress-induced neuronal oxidative stress [28,31]. In the present work, rats exposed to immobilization stress exhibited anxiety-like symptoms by decreasing time spent in an open arm (Figure 2) and light compartment (Figure 3). Similarly, depression-like symptoms were exhibited by increased immobility time while decreasing swimming and climbing time in FST (Figure 4). It is believed that free radical production at the central and periphery level deteriorates the anatomy of the cell and produces peroxidation of the cell membrane’s lipid contents [43]. Damage of neuronal cells leads to physiological malfunction of the brain, which may be linked with various behavioral deficits (anxiety and depression). Extensive studies also relate stress-induced oxidative stress to neuroinflammation. It is reported earlier that a stress situation also provokes the synthesis of inflammatory cytokines [44], which may worsen the normal situation and alter the behavioral responses. The activation of inflammatory events following physical and psychological stress burden along with the environmental stress produces cytokines and other inflammatory mediators, which are involved in the development of behavioral impairment and neurodegenerative disorders [45]. Therefore, anxiety-/depression-like behavior observed in the present study is due to increased oxidative stress markers (Figure 6) and inflammatory responses in rats experienced to immobilization stress. It is apparent that repeated administration of *M. madrespatana* peel powder dose-dependently increased exploratory activity in the novel and familiar environment in normal/unstressed animals (Figure 1). Moreover, the selected dose of *M. madrespatana* exhibited increased time spent in lightbox and open arm of LDA (Figure 2) and EPM (Figure 3), respectively, and decreased immobility and increased swimming and climbing time (Figure 4) in both unstressed and stressed animals. It is indicated that the selected dose of *M. madrespatana* increased motor behavior in unstressed animals (Figure 1, Figure 2 and Figure 3) as well as in stressed (Figure 2 and Figure 3) animals, and produced anxiolytic and antidepressant effects. Increased oxidative stress stimulates the hypothalamic-pituitary-adrenal axis (HPA-axis), which causes the further release of glucocorticoids [46]. The enhanced release of glucocorticoids may also involve stress-induced behavioral alteration [46,47].

It is well documented that an imbalance between pro-oxidant and antioxidant causes various diseases such as anxiety and depression [48]. The natural cellular antioxidant enzyme SOD, CAT, and GPx fight against ROS production [49] and produce protective effects against various threats/diseases [50]. The present study also showed that single immobilization stress imbalanced the normal natural environment at the cellular level and increased lipid peroxidation/malondialdehyde (Figure 6) and decreased antioxidant enzymes (SOD, CAT, and GPx, Figure 7). Numerous studies also showed free radical production become enhanced and the antioxidant mechanism becomes suppressed in anxiety and depression in humans [40] as well as in animal studies [51]. Various treatments are available, which produce anxiolytic and anti-depressive effects by increasing the antioxidant mechanism and diminishing oxidative stress markers. The available treatment, which is reported until the date are 5-HT-1A agonist such as buspirone [47], 8-OH-DPAT [52], etc. Various parts of plants such as *Allium cepa* peel [31], Banana fruit pulp, and peel [51], etc., and an active compound, which are present in various plants such as quercetin [40], gallic acid [42], etc. Previously, it was reported that leaf extract of *M. madrespatana* have anxiolytic [53] anti-inflammatory [54] and antioxidant [53] effects. Having potent antioxidant *M. madrespatana* enhanced free radical scavenging activity and improved the antioxidant enzyme system [53,54]. The present study showed that *M. madrespatana* peel powder improves immobilization stress-induced antioxidant enzymes by increasing SOD, CAT, and GPx (Figure 7) and decreasing MDA levels (Figure 6). These results indicating that single immobilization stress-induced oxidative stress is protected by repeated administration of *M. madrespatana* peel powder possibly via its antioxidant effects.

Serotonin neurotransmission is an important element of the stress response [55]. It was reported earlier that acute stress enhances 5-HT function [40] and increases 5-HT levels in the whole brain [31,40] and various regions of the rat’s brain (such as hippocampus, [56]). Dysfunction of serotonergic neurotransmission produces various psychiatric ailments including anxiety [57], depression [58], and cognitive impairment [59]. Increased levels of 5-HT enhancing cognitive function [31,40] and exhibiting anti-depressive behavior [60], however, reduced concentration of 5-HT in the brain [30], and in the hippocampus [33] by altering memory function. Results of the present study showed that the administration of *M. madrespatana* decreased 5-HT and 5-HIAA levels in immobilized rats than water-treated rats (Figure 9) and proved its anxiolytic property. Apart from that, the reduction of 5-HT and its metabolite was smaller in *M. madrespatana*-treated stressed animals than unstressed animals (Figure 9). In the present study, 5-HT turnover was found comparable in *M. madrespatana*-treated unstressed animals and streamed animals (Figure 9). Although memory improvement (Figure 5) was found in both *M. madrespatana*-treated stressed rats and unstressed rats, it was more improved in *M. madrespatana*-stressed rats than unstressed rats, which may be due to a smaller decrease of 5-HT and 5-HIAA levels in *M. madrespatana*-stressed animals (Figure 9). Similarly, the anti-depressive effect of *M. madrespatana* was observed in both stressed and unstressed rats. It is reported earlier that the whole plant of *M. madrespatana* contains an alkaloid, terpenoid, flavonoid, and phenolic compounds [61] and the potential role of *M. madrespatana* as an antioxidant is due to the presence of these phytochemicals, which are involved in its anxiolytic, antidepressant, and memory-enhancing effects.

AChE is a degradative enzyme and a biomarker to determine the cognitive function. ACh is a key neurochemical, which is concerned with memory and learning [62]. Previously, it has reported that acute stress increases the release of ACh in the hippocampus [63] and modulates the genes that regulate ACh availability after stress and blockade of AChE [64]. Single immobilization increases the ACh availability in the synapse and enhances memory function [64] as observed increased levels of ACh in our study (Figure 10). The previous report showed that single immobilization stress decreased AChE activity [31] and decreased latency escape, which signifies improved memory in these rats. The present result on AChE activity (Figure 8) and ACh levels (Figure 10) are consistent with the previous report [31,40]. Although acute immobilization stress increased oxidative stress (Figure 5), memory improvement as observed in the present study may be due to increased cholinergic function and antioxidant effect of M.M. Previously, various phytochemicals such as gallic acid [42], quercetin [40], and curcumin [65] decreased AChE activity and increased the availability of ACh with improved memory function. It may be postulated that M.M. peels involved in neuronal plasticity by its free radical scavenging potential increase the ACh levels (Figure 10), and produce an inhibitory effect on AChE activity (Figure 8), as observed in the present results. Single immobilization stress and *M. madrespatana* peel powder have the potential to improve cognitive function via its antioxidant effects, which is the novelty of the present study.

## 5. Conclusions

It is concluded that the present research supports the antioxidant potential of *M. madrespatana* as reported previously, which protects against single immobilization stress-induced anxiety and depression-like behaviors in rats. Besides, *M. madrespatana* also involved in the regulation of serotonin function and produces anxiolytic, antidepressant, and memory-enhancing effects. These consequences offer the pharmacological indication of folkloric practices of this plant for some neurological disorders. It is suggested that daily intake of *M. madrespatana* peel could be an enhanced antioxidant system of the body and a potential medication for the daily stress-based situation. However, in the future, more studies could be performed to evaluate the effect of *M. madrespatana* on female rats and using chronic stress conditions.
