# Correction: Samad, N., et al. Behavioral and Biochemical Effects of *Mukia madrespatana* Following Single Immobilization Stress in Rats. *Medicina* 2020, *56*, 350

**DOI:** 10.3390/medicina56080391

**Published:** 2020-08-05

**Authors:** Noreen Samad, Amna Ali, Farzana Yasmin, Riaz Ullah, Ahmed Bari

**Affiliations:** 1 Department of Biochemistry, Bahauddin Zakariya University, Multan 60800, Pakistan; amnaali394@gmail.com; 2 Department of Biomedical Engineering, NED University of Engineering and Technology, Karachi 75270, Pakistan; farzanayasminpk@yahoo.com; 3 Department of Food Engineering, NED University of Engineering and Technology, Karachi 75270, Pakistan; 4 Department of Pharmacognosy (MAPPRC), College of Pharmacy, King Saud University, Riyadh 12372, Saudi Arabia; rullah@ksu.edu.sa; 5 Central Laboratory, College of Pharmacy, King Saud University, Riyadh 12372, Saudi Arabia; abari@ksu.edu.sa

## Change in Funding part:

We would like to change the funding part of paper [1] from:

**Funding:** The authors extend their appreciation to the Research Supporting Project Number (RSP2019/110), King Saud University Riyadh, Saudi Arabia for financial support.

to the correct version, as follows:

**Funding:** The authors extend their appreciation to the Research Supporting Project Number (RSP2020/110), King Saud University Riyadh, Saudi Arabia for financial support.

We apologize for any inconvenience caused to the readers.
